# Role of Anesthetics and Their Adjuvants in Neurovascular Protection in Secondary Brain Injury after Aneurysmal Subarachnoid Hemorrhage

**DOI:** 10.3390/ijms22126550

**Published:** 2021-06-18

**Authors:** Umeshkumar Athiraman, Gregory J. Zipfel

**Affiliations:** 1 Department of Anesthesiology, Washington University, St. Louis, MO 63110, USA; 2 Department of Neurological Surgery, Washington University, St. Louis, MO 63110, USA; zipfelg@wustl.edu

**Keywords:** anesthetics, anesthetic adjuvants, neurovascular protection, aneurysmal subarachnoid hemorrhage, early brain injury, delayed cerebral ischemia, neurological outcomes

## Abstract

Aneurysmal rupture accounts for the majority of subarachnoid hemorrhage and is responsible for most cerebrovascular deaths with high mortality and morbidity. Initial hemorrhage severity and secondary brain injury due to early brain injury and delayed cerebral ischemia are the major determinants of outcomes after aneurysmal subarachnoid hemorrhage. Several therapies have been explored to prevent these secondary brain injury processes after aneurysmal subarachnoid hemorrhage with limited clinical success. Experimental and clinical studies have shown a neuroprotective role of certain anesthetics in cerebrovascular disorders including aneurysmal subarachnoid hemorrhage. The vast majority of aneurysmal subarachnoid hemorrhage patients require general anesthesia for surgical or endovascular repair of their aneurysm. Given the potential impact certain anesthetics have on secondary brain injury after SAH, appropriate selection of anesthetics may prove impactful on overall outcome of these patients. This narrative review focuses on the available evidence of anesthetics and their adjuvants in neurovascular protection in aneurysmal subarachnoid hemorrhage and discusses current impact on clinical care and future investigative directions.

## 1. Introduction

The incidence of hemorrhagic stroke in the general population accounts for approximately 20% of all the strokes, with 5% due to subarachnoid hemorrhage (SAH) [1]. The morbidity and mortality remains high for this patient population. For aneurysmal SAH, 30% of patients die [2] and 50% of survivors have long-term cognitive deficits that preclude their return to work [3]. The two most important determinants of outcome after SAH are initial hemorrhage severity and secondary brain injury due to early brain injury (EBI) and delayed cerebral ischemia (DCI). EBI occurs in 12% of patients, develops 1–3 days after SAH and is characterized by blood–brain barrier (BBB) disruption, neuronal cell death, neuroinflammation and cerebral edema [4]. DCI occurs in ~30% of patients, develops 4–12 days after SAH and is characterized by large artery vasospasm, distal autoregulatory dysfunction, microvessel thrombosis and cortical spreading depression [5]. Though many strategies to prevent EBI and DCI have been explored over the years, none have proven efficacious. New therapies are desperately needed to treat these conditions.

In recent decades, many experimental studies have shown a strong neuroprotective effect of certain anesthetics on EBI and DCI. Some of these studies have also shown an impact on mortality and neurological outcome. A more limited set of clinical studies suggest that certain anesthetics may have a protective effect in SAH patients. The vast majority of aneurysmal SAH patients will be exposed to anesthetics either in the operating room for surgical repair of the aneurysm, the interventional neuroradiology suite for endovascular treatment of the aneurysm, or in the neurointensive care unit for sedation after the initial hemorrhagic insult. Given the potential protective effects of certain anesthetic agents and their adjuvants, choice of anesthetic technique may prove critical in the management of SAH patients. The aim of the present review is to critically analyze the available evidence implicating a role of certain anesthetics and their adjuvants in neurovascular protection in aneurysmal SAH.

## 2. Methodology

**Figure 1:**
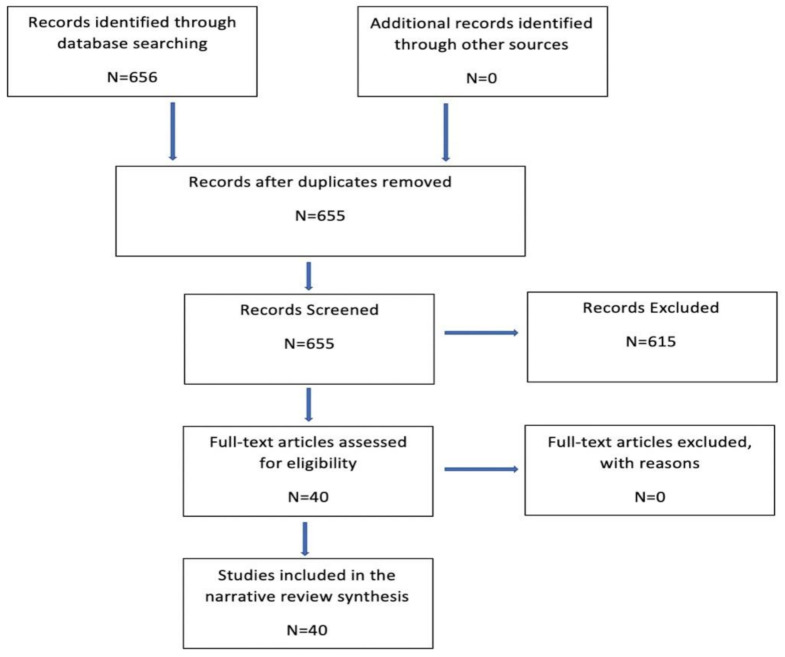
Flow diagram of search strategies.

Literature search for this review was performed for the following inhalational anesthetics (desflurane, sevoflurane, isoflurane, halothane, Xenon, Argon), intravenous anesthetics (propofol, etomidate, barbiturates, ketamine) and adjuvants for anesthetics (clonidine, dexmedetomidine, benzodiazepines and opioids) in relation to aneurysmal SAH until April 2021. Literature search was conducted in PubMed and EMBASE database. Clinical and experimental studies focusing on the anesthetic effects on EBI, DCI and neurobehavioral outcomes after aneurysmal SAH are included. Studies with abstracts only, languages other than English, with additional treatments on top of anesthetics, studies which did not directly assess one of the components of EBI or DCI and invitro studies were not included. Following search criterions were used for the review: Inhalational anesthetics (isoflurane, desflurane, sevoflurane, halothane, Xenon, Argon) and aneurysmal subarachnoid hemorrhage, intravenous anesthetics (propofol, etomidate, barbiturates, ketamine) and aneurysmal subarachnoid hemorrhage and adjuvants (clonidine, dexmedetomidine, benzodiazepines and opioids) and aneurysmal subarachnoid hemorrhage. A total of 655 articles were screened and 40 articles met the inclusion criteria which forms the basis of this narrative review (Figure 1).

## 3. EBI after SAH

EBI occurs 1–3 days after SAH and it is an independent risk factor for poor outcomes. It consists of several elements such as blood–brain barrier disruption, cell death, neuroinflammation and cerebral edema [4]. Though the incidence of EBI is less, it is increasingly recognized as one of the important contributors of significant morbidity and mortality after SAH. After the initial aneurysmal rupture causing SAH, there is a peak rise in intracranial pressure (ICP) leading to a transient global cerebral ischemia. The transient global cerebral ischemia and the hemoglobin in the subarachnoid space exacerbates the cerebral injury by activating several pathways such as apoptosis, inflammation and oxidative stress which may contribute to the blood–brain barrier disruption eventually causing cerebral edema [6]. Currently, there are no targeted therapies available to treat EBI and the primary management is focused on ICP control and cerebral perfusion pressure optimization [7]. Here, we review the available preclinical (Table 1) and clinical (Table 2) evidence from the literature on the anesthetic/adjuvants effects on EBI after SAH.

## 4. Anesthetics/Adjuvants in EBI after SAH

### 4.1. Inhalational Anesthetics

#### 4.1.1. Isoflurane

##### Experimental Studies

Altay et al. reported a series of complementary studies examining the protective effect of brief exposure to isoflurane anesthesia on SAH-induced EBI [8,9,10] They showed in an endovascular perforation mouse model of SAH that exposure to 2% isoflurane for 1hr when started 1 h post-SAH attenuated brain water content, decreased neuronal apoptosis and improved neurological score at 24 hr [8]. These protective effects, however, were not seen at 72 h [8]. Through a series of pharmacologic experiments, this EBI protection was causally linked to the sphingosine pathway. In this study, isoflurane exposure was shown to increase sphingosine kinase 1 (SphK1) expression, Akt phosphorylation and decreased cleaved caspase-3 after SAH. Administration of specific SphK antagonist, N, N dimethyl sphingosine (DMS) and a S1P1-3 receptor antagonist, VPC23019 eliminated the beneficial effects of isoflurane against EBI indicating that isoflurane’s neuroprotective effect was mediated through sphingosine related pathway. Subsequent studies by the same authors validated the protective effect of brief exposure to 2% isoflurane (but not 1% isoflurane) against EBI, including attenuation of blood–brain barrier disruption^9^ and reduction in neuroinflammation [10]. In both these studies, isoflurane’s EBI protection was reversed by DMS and VPC23019 representing the involvement of sphingosine pathway in isoflurane’s neuroprotection.

##### Clinical Studies

A recent prospective study [11] examined the effects of multiple anesthetics (isoflurane, sevoflurane, desflurane, propofol) at different time points (baseline, one hour after anesthetic induction, one hour after end of anesthesia) on cerebrospinal fluid (CSF) and serum caspase-3 levels in SAH patients undergoing aneurysmal coiling/clipping. A significant reduction in CSF caspase-3 levels and an increase in serum caspase-3 levels was noticed at one hour after anesthetic exposure, which was consistent with all anesthetics. The authors concluded that anesthetics may have a transient resuscitative role during acute brain injury after SAH by potentially reducing caspase-3 expression/activity in the brain injury, as reflected by a reduction in caspase-3 levels in CSF.

#### 4.1.2. Sevoflurane

##### Experimental Studies

Sevoflurane exposure in an endovascular perforation mouse model of SAH was shown to protect against EBI. The authors administering varying concentrations of sevoflurane (1.5%, 3%, 4.5%) one hour after SAH induction at three different treatment durations (30, 60, 90 min) showed that 1.5% sevoflurane for 60 min as well as 3% for 30 or 60 min provided significant protection against EBI including improved neurological scores, less cerebral edema and neuronal apoptosis [12]. These results indicate proper sevoflurane dosing is critical towards achieving maximal EBI protection, with higher concentration (4.5%) and prolonged exposure (90 min) resulting in loss of neurovascular protection. No data was provided regarding underlying mechanism for the demonstrated EBI protection. A subsequent study by Altay et al., [13] comparing the effects of 3% sevoflurane vs 2% isoflurane in a mouse endovascular perforation SAH model showed that both anesthetics attenuated SAH-induced EBI to a similar level and also improved neurological function at 24 h. Though the mechanism beyond the anesthetic protection was not explored in the study, the authors noted that both anesthetics upregulated sphingosine kinase-1 protein expression suggesting a possible involvement of the Sphk1 related pathway in anesthetic induced EBI protection. A recent study by Beck-Schimmer et al. comparing sevoflurane vs propofol sedation in a rat endovascular perforation SAH model provided evidence that sevoflurane sedation post SAH attenuated cerebral edema [14]. While a causal mechanism behind sevoflurane’s protection had not been explored in the study, authors speculated that sevoflurane might attenuate cerebral edema by stabilizing the adherens junction protein beta catenin.

### 4.2. Inert or Noble Gases

#### Experimental Studies

Helium, neon, argon, krypton and xenon are considered inert or noble gases, which at standard temperature and pressure have very low anesthetic activity. Xenon is the only inert gas that can be used as an anesthetic at atmospheric pressure, though it is not clinically used due to its expense and technical complexities. Hollig et al. [15] showed that *rats* exposed to a mixture of 50 vol% argon/50 vol% oxygen (O_2_) for 1 h beginning 1 h after SAH induction in an endovascular perforation model maintains animal body weight and reduces mortality when compared to control. However, neuroscore and cerebral edema did not differ between groups. The authors noted a significant increase in hypoxia inducible factor—1 alpha (HIF-1α) and hemeoxygenase—1 (HO-1) expression by argon exposure, 24 h after SAH and speculated that argon’s neuroprotective effect was mediated through HIF-1α induced HO-1 expression. Veldeman et al [16] showed that rats exposed to Xenon (50 vol% O_2_/50 vol% Xe) for 1 h initiated at 1 h post SAH in an endovascular perforation model protected against hippocampal damage including reduced neuronal damage and microglial activity in the CA3 and dentate gyrus. A subsequent study administering Xenon to rats through echogenic liposomes (Xe-ELIP) (600 μL per animal, 10 mg lipid/mL) 30 min after SAH in an endovascular perforation model reduced hemorrhage volume and neuronal cell death, improved neurological function and decreased mortality [17]. In all studies examining inert or noble gases, no data were provided to causally relate any underlying mechanism for the demonstrated EBI protection.

### 4.3. Intravenous Anesthetics

#### 4.3.1. Propofol

##### Experimental Studies

Shi et al. reported that rats administered intravenous propofol (10 vs 50 mg/kg) beginning at 2 h and repeated 12 h after SAH in an endovascular perforation model significantly reduced cerebral edema, blood–brain barrier breakdown and improved neurological scores. A dose dependent effect of propofol was reported in the study with higher propofol dose (50 mg/kg) being more effective in improving neurological scores, reducing brain edema and BBB breakdown and lower dose of propofol (10 mg/kg) was more effective in attenuating the inflammation. Propofol was shown to exert its neuroprotective effect through activation of anti-oxidative nuclear factor erythroid-related factor 2 signaling, inhibition of pro-inflammatory Nuclear factor-kappa B (NF-kB) pathway and downregulating aquaporin, matrix metalloproteinase and cyclooxygenase-2 [18]. A follow up study by the same authors showed that intraperitoneal administration of propofol 50 mg/kg at 2 h and repeated at 12 h after SAH in a rat endovascular perforation model reduced cerebral edema, blood–brain barrier permeability and improved neurological scores. Propofol’s EBI protection in this study was reversed by LY294002, a specific inhibitor of Phosphatidylinositol 3-kinase/Akt (PI3K/Akt) signaling pathway, suggesting a possible involvement of PI3K/Akt pathway in propofol’s protection [19].

##### Clinical Studies

A prospective study where patients receiving either propofol or sevoflurane for anesthetic maintenance after temporary clip removal during intracranial aneurysm procedure observed a decrease in serum markers of oxidative stress (hydroxyl radicals and 8-isoprostane) and an increase in serum antioxidant levels (gamma-tocopherol and superoxide dismutase activity) in patients treated with propofol vs. sevoflurane. This effect was noticed from immediate postoperative period to seven days post procedure. In addition, cognitive function was better in the propofol treated group when measured by Mini-Mental State Examination (MMSE) and Montreal Cognitive Assessment (MoCA) scores up to 7 days after surgery. The authors proposed that propofol post-conditioning may protect against oxidative stress injury by enhancing antioxidation and decreasing free radical injury [20].

### 4.4. Anesthetic Adjuvants

#### 4.4.1. Dexmedetomidine

##### Experimental Studies

Dexmedetomidine, a highly selective alpha 2 adrenergic receptor agonist, is a sedative with analgesic and anesthetic sparing effects. Intraperitoneal administration of dexmedetomidine (25 μg/kg) in rats immediately and 2 h post SAH in an endovascular perforation model reduced cerebral edema, blood–brain barrier permeability and improved neurological scores. Dexmedetomidine administration was also shown to increase the activated extracellular signal-regulated kinase (ERK) after SAH. PD98095, an inhibitor of ERK activation abolished this effect and also reversed dexmedetomidine’s EBI protection causally linking dexmedetomidine’s neuroprotection in SAH to ERK signaling pathway [21]. Similar results were reported by Yin et al., who noted that intraperitoneal administration of dexmedetomidine (25 μg/kg) in rats, 2 h post SAH in an endovascular perforation model reduced cerebral edema, neuronal apoptosis, blood–brain barrier permeability, neuroinflammation and improved neurological scores. In this study, dexmedetomidine’s protection against EBI was proposed to mediate via inhibition of nucleotide-binding oligomerization domain-like receptor family pyrin domain-containing 3 inflammasome and Toll-like receptor 4/NF-kB pathway [22].

#### 4.4.2. Opioids

##### Experimental Studies

Sun et al., [23] noted that the administration of salvinorin A (a selective kappa opioid receptor agonist) attenuated cerebral edema (as measured by the volume of the lateral ventricle by MRI on day 3 after SAH) in a rat endovascular perforation SAH model. In addition, authors showed that salvinorin A reduced neuronal apoptosis in hippocampus. Through pharmacological experiments, this EBI protection of salvinorin A was linked to PIK3/Akt pathway.

## 5. DCI after SAH

DCI is another important independent risk factor for poor patient outcome after SAH. Cerebral vasospasm is an important contributor for DCI and for decades, it was attributed to be the primary driver of poor outcomes [24] Recent evidence showing that attenuation of cerebral vasospasm did not lead to a significant change in functional outcome or mortality [25], indicating that the pathophysiology underlying DCI is multifactorial and that other pathophysiological factors, which are independent of vasospasm, can contribute to the outcome. The other contributors of DCI which are causally linked to the patient outcomes are microvascular dysfunction, microvascular thrombosis and cortical spreading depression.^5^ Some overlap between the mechanisms of EBI and DCI injury exists, such as the oxidative stress and inflammation. Additional mechanisms, such as increase in procoagulant activity leading to microvessel thrombosis, are noted in DCI patients [24]. Several treatment strategies for DCI have failed in the past with only a limited success with nimodipine [24,26]. The treatment failures may have resulted likely from targeting only a single element of what has proven to be a multifactorial process [24,26]. Here, we review the available preclinical (Table 3) and clinical (Table 4) evidence from the literature on the anesthetic/adjuvants effects on DCI after SAH.

## 6. Anesthetics/Adjuvants in DCI after SAH

### 6.1. Inhalational Anesthetics

#### 6.1.1. Isoflurane

##### Experimental Studies

The first experimental study examining the impact of anesthetic conditioning on DCI came from Milner et al., who exposed wild-type mice to a brief period of isoflurane anesthesia (2% isoflurane for 1 h) at several time points after induction of SAH via endovascular perforation technique. They found that isoflurane conditioning initiated 15min, 1hr and 3 h (but not 6 h) after SAH significantly improved multiple elements of DCI including large artery vasospasm, microvessel thrombosis and autoregulatory dysfunction and that this neurovascular protection led to improved neurological outcome [27]. Through a series of pharmacological and genetic interventions, this DCI protection was causally linked to endothelial cell-derived hypoxia inducible factor—1 alpha (HIF-1α). In this study, isoflurane was shown to modulate HIF transcriptional targets such as BCL2 Interacting Protein 3, erythropoietin and glucose transporter 1. Genetic and pharmacologic inhibition of HIF-1α blocked these changes and eliminated the isoflurane’s protection against SAH-induced DCI indicating that isoflurane induced DCI protection is mediated through HIF-1α. Subsequently, several additional studies have similarly shown in mouse models of SAH that isoflurane conditioning provides protection against DCI and neurological deficits and that this protection is HIF-1α-mediated [28,29,30]. More recently, Athiraman et al. [31] provided evidence showing that a brief exposure of 2% isoflurane for one hour initiated one-hour post SAH in a mouse endovascular perforation model attenuated neurovascular deficits caused by SAH. In this study, isoflurane was shown to upregulate endothelial nitric oxide synthase (eNOS) expression and the DCI protection was causally linked to eNOS through pharmacologic and genetic interventions. In all the above-mentioned studies, concentration of isoflurane employed for the experiments was 2%, which is a supratherapeutic dose and not commonly used for the patients during surgical procedures. Therefore, a subsequent study by Athiraman et al., [32] comparing three different doses of isoflurane (subanesthetic (0.5%), anesthetic (1%) and a supratherapeutic dose (2%)) in a mouse endovascular perforation SAH model provided interesting observation showing that all three doses of isoflurane afforded significant protection against SAH-induced DCI and no difference in neurovascular protection was appreciated between three doses. No mechanism was explored in this study. A follow up study by Liu et al. [33], elucidating the mechanism by which isoflurane conditioning provides DCI protection, showed that isoflurane significantly downregulated SIRT1 (silent mating type information regulation 2 homolog) expression and co-administration of a highly selective SIRT1 inhibitor (EX-527) with isoflurane did not block isoflurane induced DCI protection. This indicates that SIRT1 may not be involved in the molecular cascade of isoflurane conditioning induced DCI protection. Nevertheless, authors revalidated their initial findings showing that a brief exposure of 2% isoflurane provided significant DCI protection.

#### 6.1.2. Sevoflurane and Desflurane

##### Clinical Studies

The first hint that inhalational anesthetics may provide protection against vasospasm and/or DCI in SAH patients came from Wang et al. [34], who examined plasma concentrations of endothelin-1 (ET-1)—a major contributing molecule to vasospasm pathophysiology—in SAH patients undergoing aneurysm clipping. They found that desflurane anesthesia reduced ET-1 levels at several different time points during aneurysm surgery as compared to pre-induction ET-1 levels. Additional evidence that inhalational anesthetics may have a protective effect on vasospasm came from Lee et al., who reported that SAH patients undergoing desflurane anesthesia during aneurysm clipping suffered a lower incidence of transcranial Doppler-evident vasospasm as compared to SAH patients undergoing propofol anesthesia [25]. No difference in angiographic vasospasm or DCI, however, was noted [35]. The most direct evidence to date suggesting inhalational anesthetics provide protection against vasospasm and DCI in SAH patients came from our study where we examined a cohort of patients who underwent inhalational anesthetic only technique (sevoflurane or desflurane) vs. combined anesthetic technique (sevoflurane or desflurane plus propofol) during surgical or endovascular aneurysm repair. We found that the inhalational anesthetic only technique was associated with lower incidence of angiographic vasospasm and that desflurane anesthesia in particular had a potential protective effect against DCI [36]. A follow-up retrospective study by our group in a larger cohort of SAH patients showed that inhalational anesthetic exposure reduces both angiographic and symptomatic vasospasm [37]. However, it is important to note that no impact on functional outcome at discharge was found in both studies as measured by modified Rankin scale (mRS). More recently, a preliminary prospective study comparing the effects of desflurane vs propofol on post-operative cognitive dysfunction (POCD) in SAH patients undergoing aneurysm clipping procedure did not find a difference in the POCD incidence between the groups [38].

### 6.2. Intravenous Anesthetics

#### 6.2.1. Propofol

##### Clinical Studies

A prospective study of SAH patients undergoing aneurysm clipping conducted by Luo et al. examined the potential effects of propofol anesthesia on cerebral vasospasm by measuring intra-operative levels of calcitonin gene related peptide (CGRP)—a well described vasodilator with mechanistic links to cerebral vasospasm—during aneurysm surgery. They found that propofol significantly reduced intraoperative CGRP levels at several different time points, which might suggest propofol anesthesia may potentially increase the risk of cerebral vasospasm after SAH [39]. In contradistinction, Hertle et al. [40] provided evidence that propofol anesthesia may provide a protective effect against one element of DCI known as cortical spreading depolarization. They performed a multicenter observational study in brain-injured patients including those with SAH and examined the impact of several analgesics and sedatives on cortical spreading depolarization as measured by spreading depolarization, isoelectric spreading depolarization and spreading depolarization clusters. Propofol was found to reduce the occurrence of spreading depolarization clusters, though the underlying mechanism behind this protection was not explored. Recently, two preliminary prospective studies examining the impact of propofol during aneurysm clipping in SAH patients did not find an effect on cognitive function (as measured by a modified form of MMSE and MoCA scores) at the time of hospital discharge [38,41].

#### 6.2.2. Ketamine

##### Clinical Studies

A retrospective study conducted by Brelie et al [42] examined the effects of ketamine vs non-ketamine sedation on the occurrence of DCI-related cerebral infarctions in ventilated SAH patients. They found the rate of DCI-associated infarctions was lower in those treated with ketamine. The postulated underlying mechanism accounting for this potential protective effect included (1) suppression of cortical spreading depolarization, (2) NMDA antagonism and (3) a direct vasodilatory effect. In addition, several studies have examined the impact of ketamine on cortical spreading depression in SAH patients. Sakowitz et al. [43] showed in one SAH patient that ketamine inhibited spreading depolarization and also restored the regular pattern electrocorticographic activity. The aforementioned study by Hertle et al. found that ketamine reduced the occurrence of all three measurements of cortical spreading depolarization [40]. Additional evidence for ketamine’s protective effect on spreading depolarizations came from Carlson et al. [44], who showed that ketamine significantly inhibited cortical spreading depolarization over wide range of doses (.55 mg/kg/h–1.15 mg/kg/h) with maximum effect seen at or above 1.15 mg/kg/h. More recently, Santol et al. [45] provided evidence showing that sedation with s-ketamine in the mechanically ventilated SAH patients was associated with reduced incidence of spreading depolarizations.

### 6.3. Anesthetic Adjuvants

#### 6.3.1. Clonidine

##### Experimental Studies

Clonidine is a relatively nonspecific α2 adrenergic receptor agonist with sedative, analgesic and anesthetic sparing effects. Bunc et al. showed that intramuscular administration of a clinically relevant dose of clonidine (0.03 mg/kg) immediately after induction of SAH and continued daily until animal sacrifice prevented basilar artery vasospasm in a rabbit cisterna magna injection model. Though the underlying mechanism was not specifically explored, its anti-vasospastic effect was attributed to clonidine’s ability to block the sympathetic nervous system activity [46].

#### 6.3.2. Dexmedetomidine

##### Experimental Studies

Ayoglu et al. [47] compared the effects of two different doses of dexmedetomidine (5 and 10 μg/kg) administered intraperitoneally 1 h after SAH and repeated 24 h later in a rat cisterna magna injection model. They found a dose-dependent effect of dexmedetomidine against SAH-induced vasospasm. This DCI protection was postulated to be the result of reduced oxidative stress and blockade of the sympathetic nervous system. Similar results were noted by Song et al. [48], who found that 10 μg/kg dexmedetomidine administered intraperitoneally 1 h after SAH and repeated 24 h later reduced vasospasm and improved neurological outcome in a rat cisterna magna injection model. Given that dexmedetomidine also reduced CSF levels of interleukin-6, the authors hypothesized that the DCI protection was related to its anti-inflammatory action.

##### Clinical Studies

A recent correspondence from Esfahani et al., [49] investigating SAH patients who received dexmedetomidine for ICU sedation did not find an association with vasospasm protection or favorable clinical outcomes at discharge as measured by mRS and Glasgow Outcome scale. As noted by the authors, the study was limited by small sample size, non-standardized dexmedetomidine dosing and difference in the clinical severity between the compared groups. In contradistinction, Okazaki et al., [50] in their retrospective study showed that SAH patients who received low dose of dexmedetomidine (0.01–0.20 μg/kg/h) during the first 24 h of admission were associated with favorable neurological outcomes at discharge as measured by mRS scale.

#### 6.3.3. Benzodiazepines

##### Clinical Studies

In the Hertle et al. [40] study, the impact of a midazolam (a sedative and anxiolytic) on cortical spreading depolarization in brain-injured patients was examined. They noted that midazolam sedation may actually increase the occurrence of cortical spreading depolarization. The proposed hypothesis for this effect was GABAergic inhibition leading to suppression of brain metabolism and exacerbation of energy dependent ion pump failure. One other small retrospective study by Hertle et al. [51] showed that sedation with gamma aminobutyric acid related drugs (flunitrazepam, midazolam and propofol) in the mechanically ventilated SAH patients was associated with poor outcomes as measured by Glasgow outcome scale at 6 months.

## 7. Summary of Anesthetics/Adjuvants and Neurovascular Protection in SAH

### 7.1. Inhalational Anesthetics

The most studied inhalational anesthetic in preclinical studies has been isoflurane. Several experimental studies have shown that isoflurane provides significant protection against both EBI and DCI after SAH [8,9,10,13,27,28,29,30,31,32,33]. To date, however, isoflurane has not been examined in clinical studies of SAH. Sevoflurane—a commonly used inhalational anesthetic for neurosurgical procedures—has been shown in several preclinical studies to protect against multiple components of SAH-induced EBI such as cerebral edema and neuronal apoptosis including improved neurological outcomes at 24 h [12,13,14]. No experimental studies have yet evaluated sevoflurane’s impact on SAH-induced DCI. Finally, though no experimental studies have thus far evaluated the impact of desflurane on SAH-induced secondary brain injury (EBI and DCI), three clinical studies have been reported, all of which show desflurane likely reduces the incidence of SAH-induced vasospasm [34,35,36]. Finally, no studies have been conducted to examine the impact of inhalational anesthetics on the long-term neurobehavioral outcomes after SAH.

### 7.2. Intravenous Anesthetics

Available experimental and clinical evidence, though somewhat limited in scope, have shown that propofol protects against SAH-induced EBI [18,19]. Whether propofol provides protection against SAH-induced DCI, however, remains unclear. One clinical study demonstrated a protective effect against DCI [40], while another clinical study refuted this finding [39]. Given that propofol is one of the most commonly used intravenous anesthetic agents for induction and maintenance during neurosurgical procedures, it will be important to further investigate the potential protective effects of this anesthetic agent on SAH-induced secondary brain injury. Another intravenous anesthetic with a potential protective effect on DCI is the NMDA antagonist, ketamine. Though no preclinical studies examining its impact in SAH have been reported, several clinical studies show that ketamine is effective at reducing cortical spreading depression—one of the critical components of DCI—in SAH patients [40,42,43,44,45].

### 7.3. Anesthetic Adjuvants

Dexmedetomidine is a highly selective centrally acting alpha-2 adrenergic agonist that is commonly used as an adjunct to other anesthetic agents during neurosurgical procedures. Several experimental studies show dexmedetomidine provides significant protection against both EBI and DCI following SAH [21,22,47,48]. A clinical study supports the use of dexmedetomidine in SAH patients as it shows a favorable neurologic outcome [50] and another study finds no impact on the outcomes [49]. Benzodiazepines, specifically midazolam has been shown to associate with poor outcomes in SAH patients [40,51].

## 8. Limitations of the Current Literature

(1) All the above-mentioned preclinical studies used male animals for their experiments. Importantly, however, SAH in patients occurs more frequently in females vs. males [52] and differences in SAH outcome between males and females have been noted in preclinical studies [53]. It is therefore essential that future experimental studies include female animals to address gender as a potential biologic variable. (2) The anesthetic dose used in the majority of experimental studies may not correlate with the anesthetic dose utilized in SAH patients. Different strains, species and the age group used in the experiments further complicates this issue. (3) Several of the experimental studies did not identify a causal mechanism for anesthetic/adjuvant induced neuroprotection in SAH, which is critical in the development of drug therapeutics. (4) Limited clinical studies are available to show the impact of anesthetics on secondary brain injury and long-term neurobehavioral outcomes after SAH. These studies are further limited by their (a) retrospective nature; (b) smaller cohort size to causally show an impact on patient outcomes; and (c) lack of standardized measures to examine cognitive and long-term neurobehavioral outcomes. Hence, well designed properly controlled larger randomized prospective studies are critical in evaluating the impact of anesthetics on the secondary brain injury outcomes after SAH.

## 9. Concluding Remarks

A growing body of literature—both experimental and clinical—indicates certain anesthetics likely have substantial protective effects against both EBI and DCI following SAH [54]. From animal studies, we have learned that protection afforded by anesthetics is multifaceted including positive effects on inflammation, blood–brain barrier breakdown, cerebral edema, neuronal apoptosis, microvascular thrombi, autoregulatory dysfunction and large artery vasospasm. The pleiotropic nature of the protective effects of certain anesthetics (EBI and DCI; neurons and cerebral vessels) has a great potential for anesthetic-based therapies to ultimately translate into robust protection against secondary brain injury in SAH patients and improvement in overall neurological outcome. The excellent safety profile and relative ease at which anesthetic agents can be delivered to acutely ill patients only enhances the promise of this therapeutic approach. Studies to further elucidate the underlying mechanisms of anesthetic-induced neurovascular protection in SAH in the hopes of identifying novel and druggable therapeutic targets are warranted, as are clinical studies designed to identify the most appropriate anesthetics, optimize their dosing and rigorously test for therapeutic benefit in SAH patients.

## 10. Future Studies

Based on the breadth of experimental and clinical data implicating certain anesthetics and anesthetic adjuvants with reducing EBI and DCI and improving neurological outcome after SAH, additional studies examining the impact of these agents in SAH are warranted. In particular, studies designed to address the following would be especially impactful: (1) determining the differential benefit of various anesthetics on SAH outcome; (2) defining optimal dosing and effective therapeutic window for SAH; (3) elucidating underlying mechanisms of anesthetic-induced neurovascular protection in SAH; and (4) exploring the impact of anesthetic treatment on long-term neurobehavioral and cognitive outcomes after SAH.

## Figures and Tables

**Table 1:** Anesthetics and adjuvants in early brain injury (EBI) after SAH in experimental studies.

References	Anesthetic Agent/Adjuvants	Species	Model	Pathway	Effects
[8]	Isoflurane	Mice	Endovascular perforation	SphK, S1P1 ↑	Reduced brain water content, decreased neuronal apoptosis, improved neurological score.
[9]	Isoflurane	Mice	Endovascular perforation	SphK, S1P1 ↑	Improved neurological score, brain edema, BBB permeability and prevented the decrease in expressions of tight junction (occludin, JAM-A and claudin-5) and adherens junction (VE- cadherin) proteins.
[10]	Isoflurane	Mice	Endovascular perforation	SphK, S1P1 ↑	Improved neurological score, brain edema, BBB permeability and decreased neuroinflammation (↓ MPO, iba-1, IL-1β, TNF-α, P-selectin, ICAM-1, p-JNK and COX-2).
[12]	Sevoflurane	Mice	Endovascular perforation	-	Improved neurological scores, reduced cerebral edema and apoptosis.
[13]	Sevoflurane/Isoflurane	Mice	Endovascular perforation	SphK 1 ↑, COX-2 ↓, Caspase -3 ↓	Both anesthetics attenuated cerebral edema, cell death, improved neurobehavioral function.
[14]	Sevoflurane/Propofol	Rat	Endovascular perforation	Stabilizing Beta Catenin	Sevoflurane attenuated cerebral edema.
[15]	Argon	Rat	Endovascular perforation	HIF-1 α ↑, HO-1 ↑	Reduced mortality and functional outcome improved as measured by the weight of the animals.
[16]	Xenon	Rat	Endovascular perforation	-	Hippocampal protection, reduction in microglial activity.
[17]	Xenon	Rat	Endovascular perforation	-	reduced hematoma volume, prevented neuronal cell death, improved neurological function and decreased the mortality and morbidity.
[18]	Propofol	Rat	Endovascular perforation	Nrf2 ↑, NF-kB ↓, AQP4 ↓, MMP-9 ↓, COX-2	Reduces cerebral edema, BBB permeability, improves Neuroscore.
[19]	Propofol	Rat	Endovascular perforation	↑ PI3K/Akt	Reduces cerebral edema, BBB permeability, improves Neuroscore.
[21]	Dexmedetomidine	Rat	Endovascular perforation	ERK ↑	Reduced cerebral edema, BBB permeability, improved neuroscore.
[22]	Dexmedetomidine	Rat	Endovascular perforation	TLR4/ NF-kB ↓, NLRP3 ↓	Reduced cerebral edema, BBB permeability, neuronal apoptosis, inhibits neuroinflammation and improves neuroscore.
[23]	Salvinorin A	Rat	Endovascular perforation	↑ PI3K/Akt	Attenuated cerebral edema and neuronal apoptosis.

↑ = Activation/Increase/elevation, ↓ = Inhibition/blockade/decrease, EBI = early brain injury, BBB = blood–brain barrier, OGD = oxygen glucose deprivation, SphK = Sphingosine kinase, S1P1 = Sphingosine-1-phosphate receptor, MPO = myeloperoxidase, iba-1 = Ionized calcium binding adaptor molecule-1, p-JNK = phospho-c-Jun N-terminal kinase, IL-1β = interleukin-1 beta, TNF-α = tumor necrosis factor-alpha, ICAM-1 = intercellular adhesion molecule-1, COX-2 = cyclooxygenase-2, HIF-1 = hypoxia inducible factor, Nrf2 = Nuclearfactorerythroid-relatedfactor2, NF-kB = Nuclear factor-kappa B, AQP4 = aquaporin, MMP-9 = matrix metalloproteinase, ERK = extracellular signal—regulated kinase, PI3K/Akt = Phosphatidylinositol 3-kinase/Akt, TLR4 = Toll-like receptor 4, NLRP3 = nucleotide-binding oligomerization domain-like receptor family pyrin domain-containing 3.

**Table 2:** Anesthetics and adjuvants in early brain injury (EBI) after SAH in patients.

References	Anesthetic Agent/Adjuvants	Sample Size	Effects
[11]	Isoflurane, Sevoflurane, Desflurane, Propofol.	44	CSF caspase-3 levels ↓, serum caspase-3 levels ↑ at one hour after anesthetic exposure.
[20]	Propofol	60	Oxidative stress is reduced (gamma-tocopherol and SOD ↑, OH and 8-isoprostane ↓), Cognitive function is improved.

↑ = Activation/Increase/elevation, ↓ = Inhibition/blockade/decrease, SAH =subarachnoid hemorrhage, CSF = cerebrospinal fluid, OH = hydroxyl radical, SOD = superoxide dismutase.

**Table 3:** Anesthetics and adjuvants in delayed cerebral ischemia (DCI) after SAH in experimental studies.

References	Anesthetic Agent/Adjuvants	Species	Model	Pathway	Effects
[27]	Isoflurane	Mice	Endovascular perforation	HIF-1 α ↑	Reduces large artery vasospasm, microvessel thrombosis, autoregulatory dysfunction and improves the neurological outcome.
[28,29,30]	Isoflurane	Mice	Endovascular perforation	HIF-1 α ↑	Reduced DCI as measured by the occurrence of new cerebral infarctions in the MRI and improved neurobehavioral outcome.
[31]	Isoflurane	Mice	Endovascular perforation	eNOS ↑	Attenuated large artery vasospasm and improved neurological outcome
[32]	Isoflurane	Mice	Endovascular perforation	-	Attenuated large artery vasospasm and improved neurological outcome
[33]	Isoflurane	Mice	Endovascular perforation	SIRT1 not involved in protection	Attenuated large artery vasospasm, microvessel thrombosis and improved neurological outcome
[46]	Clonidine	Rabbit	Cisterna Magna injection	SNS ↓	Reduced chronic vasospasm.
[47]	Dexmedetomidine	Rat	Cisterna Magna injection	MDA ↓, SNS ↓	Reduced vasospasm and oxidative stress.
[48]	Dexmedetomidine	Rat	Cisterna Magna injection	IL-6 ↓	Reduced vasospasm and improved neurological function.

↑ = Activation/Increase/elevation, ↓ = Inhibition/blockade/decrease, DCI = delayed cerebral ischemia, SAH = subarachnoid hemorrhage, ET-1 = endothelin-1, HIF-1 = hypoxia inducible factor-1, eNOS = endothelial nitric oxide synthase, SIRT1 = silent mating type information regulation 2 homolog, SNS = sympathetic nervous system, MDA = malonialdehyde, IL-6 = interleukin.

**Table 4:** Anesthetics and adjuvants in delayed cerebral ischemia (DCI) after SAH in patients.

References	Anesthetic Agent/Adjuvants	Sample Size	Effects
[34]	Desflurane	45	ET-1 ↓. May reduce the risk of acute cerebral vasospasm.
[35]	Desflurane/Propofol	102	Less incidence of TCD-evident vasospasm with desflurane.
[36]	Desflurane	157	Lower incidence of DCI.
[37]	Sevoflurane/Desflurane	390	Lower incidence of angiographic vasospasm and DCI.
[38]	Desflurane/Propofol	70	No effects on POCD at the time of discharge.
[39]	Propofol	45	CGRP↓. May potentiate the risk of acute cerebral vasospasm.
[41]	Propofol	66	No effect on cognition at time of hospital discharge.
[40]	Ketamine, midazolam, propofol	31	Ketamine ↓ the occurrence of spreading depolarization’s, clusters of spreading depolarization’s and isoelectric spreading depolarizations. (NMDA ↓) Midazolam ↑ spreading depolarization clusters. (GABA ↓). Propofol ↓ clusters of spreading depolarizations.
[42]	Ketamine	65	Lower incidence of non-procedural related infarctions probably through NMDA ↓, ↓ cortical spreading depolarization and vasodilation.
[43]	Ketamine	1	NMDA ↓. Cortical spreading depolarization ↓.
[44]	Ketamine	8	↓ spreading depolarizations.
[45]	Ketamine	66	↓ spreading depolarizations.
[50]	Dexmedetomidine	161	Favorable neurologic outcome at discharge as measured by mRS.
[49]	Dexmedetomidine	127	No impact on vasospasm or clinical outcome at discharge as measured by mRS and GOS.
[51]	flunitrazepam, midazolam, propofol	29	Associated with poor outcomes at 6 months after discharge as measured by GOS.

↑ = Activation/Increase/elevation, ↓ = Inhibition/blockade/decrease, ET-1 = endothelin, TCD = transcranial Doppler, CGRP = calcitonin gene- related peptide, GABA = Gamma- aminobutyric acid, NMDA = N-methyl- D- Aspartate, POCD = post-operative cognitive dysfunction, mRS = modified Rankin scale, GOS = Glasgow outcome scale.

## Data Availability

Not Applicable.
